# Integrated genomic analysis to identify druggable targets for pancreatic cancer

**DOI:** 10.3389/fonc.2022.989077

**Published:** 2022-12-01

**Authors:** Eko Mugiyanto, Wirawan Adikusuma, Lalu Muhammad Irham, Wan-Chen Huang, Wei-Chiao Chang, Chun-Nan Kuo

**Affiliations:** ^1^ PhD Program in Clinical Drug Development of Herbal Medicine, College of Pharmacy, Taipei Medical University, Taipei, Taiwan; ^2^ Department of Pharmacy, Faculty of Health Science, University of Muhammadiyah Pekajangan Pekalongan, Pekalongan, Indonesia; ^3^ Department of Clinical Pharmacy, School of Pharmacy, College of Pharmacy, Taipei Medical University, Taipei, Taiwan; ^4^ Department of Pharmacy, Faculty of Health Science, University of Muhammadiyah Mataram, Mataram, Indonesia; ^5^ Faculty of Pharmacy, University of Ahmad Dahlan, Yogyakarta, Indonesia; ^6^ Institute of Cellular and Organismic Biology, Academia Sinica, Taipei, Taiwan; ^7^ Department of Pharmacy, Wan Fang Hospital, Taipei Medical University, Taipei, Taiwan; ^8^ Integrative Research Center for Critical Care, Department of Pharmacy, Wan Fang Hospital, Taipei Medical University, Taipei, Taiwan

**Keywords:** genomic, pancreatic cancer, drug repurposing, fulvestrant, midostaurin

## Abstract

According to the National Comprehensive Cancer Network and the American Society of Clinical Oncology, the standard treatment for pancreatic cancer (PC) is gemcitabine and fluorouracil. Other chemotherapeutic agents have been widely combined. However, drug resistance remains a huge challenge, leading to the ineffectiveness of cancer therapy. Therefore, we are trying to discover new treatments for PC by utilizing genomic information to identify PC-associated genes as well as drug target genes for drug repurposing. Genomic information from a public database, the cBio Cancer Genomics Portal, was employed to retrieve the somatic mutation genes of PC. Five functional annotations were applied to prioritize the PC risk genes: Kyoto Encyclopedia of Genes and Genomes; biological process; knockout mouse; Gene List Automatically Derived For You; and Gene Expression Omnibus Dataset. DrugBank database was utilized to extract PC drug targets. To narrow down the most promising drugs for PC, CMap Touchstone analysis was applied. Finally, ClinicalTrials.gov and a literature review were used to screen the potential drugs under clinical and preclinical investigation. Here, we extracted 895 PC-associated genes according to the cBioPortal database and prioritized them by using five functional annotations; 318 genes were assigned as biological PC risk genes. Further, 216 genes were druggable according to the DrugBank database. CMap Touchstone analysis indicated 13 candidate drugs for PC. Among those 13 drugs, 8 drugs are in the clinical trials, 2 drugs were supported by the preclinical studies, and 3 drugs are with no evidence status for PC. Importantly, we found that midostaurin (targeted PRKA) and fulvestrant (targeted ESR1) are promising candidate drugs for PC treatment based on the genomic-driven drug repurposing pipelines. In short, integrated analysis using a genomic information database demonstrated the viability for drug repurposing. We proposed two drugs (midostaurin and fulvestrant) as promising drugs for PC.

## Introduction

Pancreatic cancer (PC) is the 12th most prevalent cancer in men and the 11th most common cancer in women, with about 450,000 new cases diagnosed worldwide every year (1). The high mortality and poor prognosis are primarily due to lack of noticeable and distinctive clinical signs or biomarkers for early detection. Aggressive metastatic spread of PC contributes to the difficulty of treatment (2). Generally, PC is classified into two types: the most frequent pancreatic adenocarcinoma, which arises in the pancreas’ exocrine glands, and the less common pancreatic neuroendocrine tumor, which occurs in the pancreas’ endocrine tissue (1). Another forms of categorization is based on whether the tumors present in the entire (solid or cystic) or on the prevailing cell differentiation structure (ductal, acinar, or endocrine). Solid types include pancreatic ductal adenocarcinoma, neuroendocrine neoplasms, acinar cell carcinomas, and pancreatoblastomas. Mucinous cystic neoplasms, intraductal papillary mucinous neoplasms, and strong pseudopapillary neoplasms are some of the less harmful cystic forms (3).

The options of PC treatment are very restricted and highly dependent on the stage of the disease. Chemotherapy treatment remains the primary choice for patients with advanced and metastatic tumors. Radiation is another treatment for unresectable, metastatic cancer when combined with chemotherapy (4). The National Comprehensive Cancer Network (NCCN) and the American Society of Clinical Oncology (ASCO) recommend modified FOLFIRINOX, gemcitabine and capecitabine, and single-agent gemcitabine or fluorouracil. S-1, an oral 5-fluorouracil prodrug as a standard treatment for PC. However, those treatments only marginally prolong life expectancy by approximately 3% (5). Furthermore, patients with PC are typically diagnosed at advanced stage with remote metastases. Therapeutic resistance is a persistent challenge, dumping therapeutic efficacy and prognosis of PC (6). Therefore, it is urgent to develop more non-surgical therapeutic approaches to effectively treat PC. One of the strategies to tackle those issues is by utilizing the available drugs for new indications.

**Figure 1:**
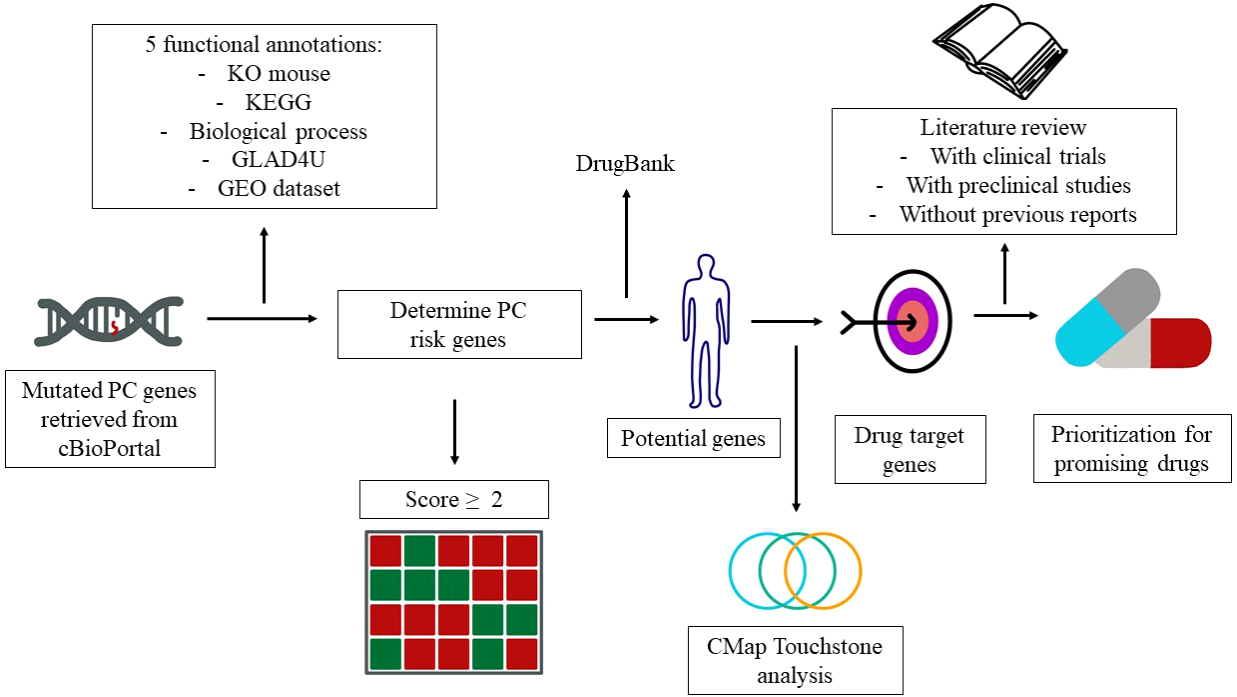
Study design and genes that contribute to pancreatic cancer (PC). The workflow elucidates the PC risk genes and candidate drugs derived from the public databases.

Drug repurposing is a method to identify new indications of a drug (7). This approach has several advantages in the drug discovery for specific indications. First, the risk of failure is lower because the repurposed drugs are relatively safe if early-stage studies have been completed in animal models and humans. Second, this approach is able to reduce the time for pharmacokinetic and toxicological studies. Third, less capital is required (8). Genomic information has been widely utilized for drug repurposing. One of the databases that provide genomic information is cBioPortal. cBioPortal is an open-access resource for the interactive exploration of multidimensional cancer genomics datasets (9). Herein, we employed cBioPortal as a main resource and integrated different annotations to prioritize the PC risk genes. The detailed flowchart of genomic-driven drug repurposing is illustrated in **Figure 1**.

## Methods

### Retrieving pancreatic cancer associated genes

PC genes with somatic mutation (PC-associated gene) were retrieved from the cBioPortal (https://www.cbioportal.org/) database (9). PC-associated genes were provided by cBioPortal, which were extracted from nine studies with 1,023 samples (**Supplementary Table 1**).

### Identifying biological pancreatic cancer risk genes

Subsequently, five functional annotations: (1) Kyoto encyclopedia of genes and genomes (KEGG); (2) Biological process (BP); (3) Knock out mouse (KO); (4) Gene list automatically derived for you (GLAD4U); and (5) Gene Expression Omnibus Dataset (GEO) were utilized to build a scoring system to prioritize PC-associated genes. The first functional annotation was KEGG to determine the molecular pathway. The second was gene ontology BP to identify genes involved in the biological protein network. The third was KO mouse to investigate whether the gene contributes to specific phenotype disease in the mouse. The fourth was GLAD4U to analyze which gene is related to a particular disease (10) , and the fifth applied four datasets from the GEO database to highlight up-regulated genes in PC samples. Only data from human PC patients was collected from each dataset for investigation. The dataset we retrieved from the GEO database were GSE28735, GSE15471, GSE16515, and GSE19650 and were depicted in **Table 1**. We used Within-Array Normalization to normalized each dataset before extracting the differential expression genes (DEGs). The DEGs were found by setting an adjusted p-value cut-off of 0.05 and log fold cut>2 (**Supplementary Figure 1)**. The annotation applied for this study was from the WebGestalt 2019 database (http://www.webgestalt.org/), a popular tool for the interpretation of gene lists derived from large-scale-omics studies (11) and GEO database (12). The significant result for annotation utilizing WebGestalt was set at a false discovery rate (FDR) < 0.05. The scoring system was utilized in a previous study by Okada et al., which identified potential treatments for rheumatoid arthritis (13). Genes that fulfilled two or more of the criteria were defined as biologicalPC risk genes.

**Table 1:** Characteristic of Gene Expression Omnibus Dataset Series.

GSE series	Platform	Total Sample	Normal	PC	PMID
GSE28735 GSE15471 GSE16515 GSE19650	GPL6244 GPL570 GPL570 GPL570	130 78 52 22	61 42 16 7	69 36 36 15	27197190 28881803 19732725 20955708

### Overlapping drug targets for pancreatic cancer with DrugBank

To uncover therapeutic candidates for repurposing in PC, we mapped biological PC risk genes to DrugBank databases (https://go.drugbank.com). The DrugBank database is a bioinformatics and cheminformatics database that provides detailed information on drug compounds and gene targets to the drug discovery and clinical medicine communities (14). Drugs having pharmacological activity, human effectiveness, approved annotations, clinical trials (http://www.ClinicalTrials.gov), and experimental drugs were among the factors utilized to query the databases.

### Prioritizing candidate drugs

The core principle behind Connectivity Map (CMap) Touchstone analysis is to compare a drug-specific gene expression profile with a disease-specific gene signature using a reference database (15). We used the CMap Touchstone database tool (https://clue.io) to grade drugs as shown in a connectivity score to prioritize the drugs for PC repurposing (-100 to 100) (16). Here, gemcitabine was used as a standard treatment for PC according to the NCCN guideline (5). The CMap database provides nine cell lines, namely A375, A549, HA1E, HCC515, HEPG2, HT29, MCF7, PC3, and VCAP. However, by using gemcitabine as the gold-standard, only three cell lines (A549, MCF7, and PC3) are available to match with gemcitabine in the CMap database.

According to the DrugBank database, the target protein of gemcitabine is the ribonucleoside-diphosphate reductase large subunit (RRM1). Thus, CMap analysis generated a score that indicates the interaction strength between candidate drugs and the protein. The ranked candidate drug was prioritized based on the score (≥ 80). Finally, a PubMed literature review and ClinicalTrials.gov were used to evaluate the reliability of candidate drugs based on the evidence from preclinical studies and clinical trials.

### Statistical analysis

All analytic workflows were performed using R Studio version 1.3.1073 (https://www.r-project.org). DEGs were obtained from the GEO dataset (https://www.ncbi.nlm.nih.gov/gds) using the R limma package. Overrepresentation analysis (ORA) was utilized to prioritize the gene in KEGG, BP, KO mouse, and GLAD4U provided by Webgestalt 2019 (11, 17). The statistical significance was established using an FDR of 0.05. R studio were used for graphic visualization.

## Results

### Prioritizing genes associated with pancreatic cancer

**Figure 2:**
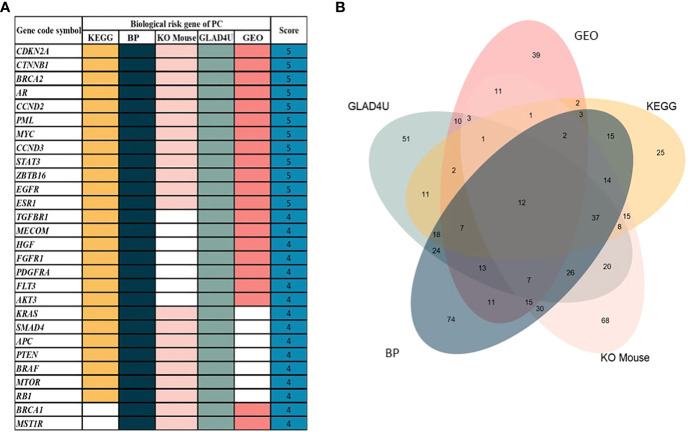
**(A)** 30 out of 318 genes after prioritization based on five functional annotations. White box indicated that no significant annotation was found within the database. **(B)** The Venn diagram shows the distribution of genes in each annotation. From 895 genes, 318 genes were identified as biological PC risk genes with accumulated scores ≥2.

We retrieved sample data from the cBioPortal database. Nine studies related to PC were obtained with 1,032 participating patients (**Supplementary Table 1**). From nine studies, 895 genes were identified as PC-associated genes (**Supplementary Table 2**). Applications of previous approaches, five functional annotations were used to generate a risk score representing the most probable candidate genes as biological PC risk genes (18–20). The result was shown in **Figure 2A** and the detailed information was in **Supplementary Table 3**. 19.32%, 34.41%, 30.17%, 27.93%, and 15.53% of PC-associated genes were found from KEGG, BP, KO mouse, GLAD4U, and GEO, respectively. Among 895 genes, 318 genes met the criteria of score ≥2 and were further defined as biological PC risk genes. The highest score (score = 5) from the five criteria was achieved by 12 genes, including *CDKN2A, CTNNB1, BRCA2, AR, CCND2, PML, MYC, CCND3, STAT3, ZBTB16, EGFR, ESR1*. Among the highest score genes, *CDKN2A* and *BRCA2* have been previously reported as dominated mutation genes in PC (21, 22).

Additionally, we analyzed the correlation among five functional annotations. Correlation coefficient analysis was conducted to assess whether the five functional annotations have possible linear relationships with each other (to avoid overlap between these functional annotations). Results showed that those five functional annotations had values of 0.30–0.50, which indicated low (weak) correlations between each other, and the results are depicted in **Supplementary Figure 2**. Additionally, the distribution score of each criterion is shown in **Figure 2B**. The number of intersection among five annotations was 12 genes. In addition, the gene number without overlapping among annotations were 25, 74, 68, 51, and 39 for KEGG, BP, KO mouse, GLAD4U and GEO, respectively.

**Figure 3:**
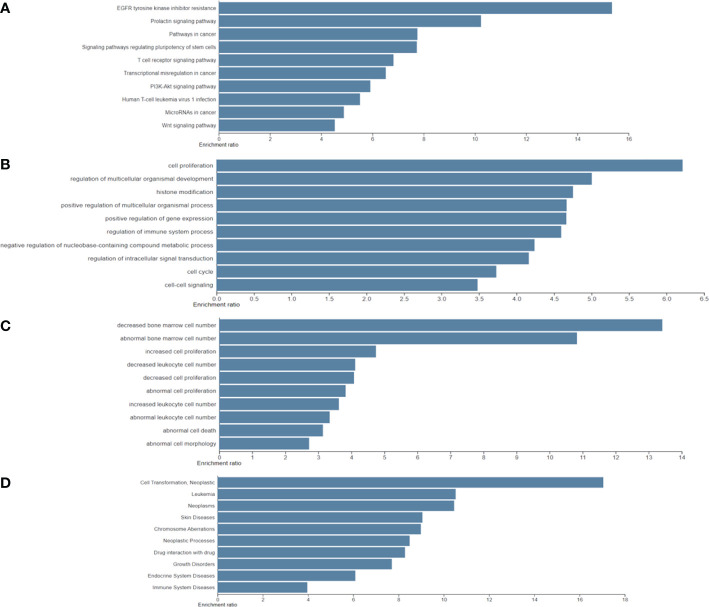
Significantly identified top 10 enriched analysis for four functional annotations: **(A)** Kyoto Encyclopedia of Genes and Genomes; **(B)** biological process; **(C)**; knockout mouse; and **(D)** Gene List Automatically Derived For You by differentially expressed genes.

Furthermore, the results of the four functional annotations, KEGG, BP, KO mouse, and GLAD4U, showed that these biological-PC risk genes are highly correspond with cancer development, especially in cell proliferation. In KEGG, the major pathway was indicated as EGFR tyrosine inhibitor resistance, prolactin signaling pathway, and pathway in cancer. In BP, the dominant biological process is in cell proliferation process. Regarding to KO mouse, results pointed out the importance of bone marrow cell proliferation. Finally, GLAD4U results suggested a strong correlation between candidate genes and neoplasms (**Figures 3A–D**). Taken together, these results indicated that 318 biological PC risk genes highly correspond to cancer growth-related signals.

### Repurposing drugs for pancreatic cancer

The next step is to map 318 biological PC risk genes into the DrugBank database. Among those genes, only 77 genes overlapped with DrugBank, indicating that not all biological PC risk genes are druggable. 77 drug target genes correspond with 216 drugs. Among the 77 target genes, over expression of EGFR has been previously identified in pancreatic tumors (23, 24) and it is associated with poor prognosis and disease progression (25, 26). Indeed, erlotinib is an EGFR tyrosine kinase inhibitor and the combination with gemcitabine demonstrated a moderate advantage (23). The results demonstrated that this process of repurposing drugs is a reasonable approach.

### Prioritization of candidate drug for pancreatic cancer

To prioritize the most potential candidate drugs for PC, 216 drugs were tested by using the CMap Touchstone database. We used the profiles of gemcitabine in the MCF7 cell line as the standard for PC treatment. Importantly, 77 drugs exhibited positive correlations. We ranked the 77 drugs according to the CMap Touchstone database scores. 13 drugs with a score>80 were defined as PC candidate drugs (**Table 2**).

**Table 2:** PC drug repurposing candidate prioritization based on CMap comparison to gemcitabine.

PC candidate drugs	Original indicator	CMap Score	Target	Level of evidence	NCT number/PubMed ID
Clomifene	Ovulation inducer	97.84	ESR1	–	PMID:25624908
Tamoxifen	Treat estrogen receptor–positive metastatic breast cancer	96.86	ESR1	Phase 2	PMID:12174927, 9641456
Fulvestrant	Metastatic breast cancer	96.06	ESR1	–	–
Raloxifene	Prevention and treatment of osteoporosis in postmenopausal women	96.04	ESR1	–	PMID:32940862
Sunitinib	Treatment of advanced renal cell carcinoma	89.59	PDGFRB	Phase 2	NCT02713763
Midostaurin	Treatment in adult patients with high-risk acute myeloid leukemia (AML)	89.03	PRKCA	–	–
Bosutinib	Treatment of chronic, accelerated, or blast-phase Philadelphia chromosome–positive (Ph+) chronic myelogenous leukemia (CML)	88.17	BCR	Phase 1	NCT01025570
Everolimus	Treatment of postmenopausal women with advanced hormone receptor-positive	87.7	MTOR	Phase 2	NCT00560963
Afatinib	Advanced or metastatic non-small-cell lung cancer (NSCLC)	86.93	EGFR	Phase 2	NCT01728818
Palbociclib	Advanced/metastatic breast cancer	84.85	CDK4	Phase 2	NCT02806648
Vorinostat	Treatment of cutaneous manifestations in patients with cutaneous T-cell lymphoma	83.12	HDAC1	Phase 1	NCT00983268
Toremifene	Treatment of metastatic breast cancer	80.82	ESR1	–	–
Axitinib	Kidney cell cancer and investigated for use/treatment in pancreatic and thyroid cancer	80.4	FLT1	Phase 3	NCT00471146

**Figure 4:**
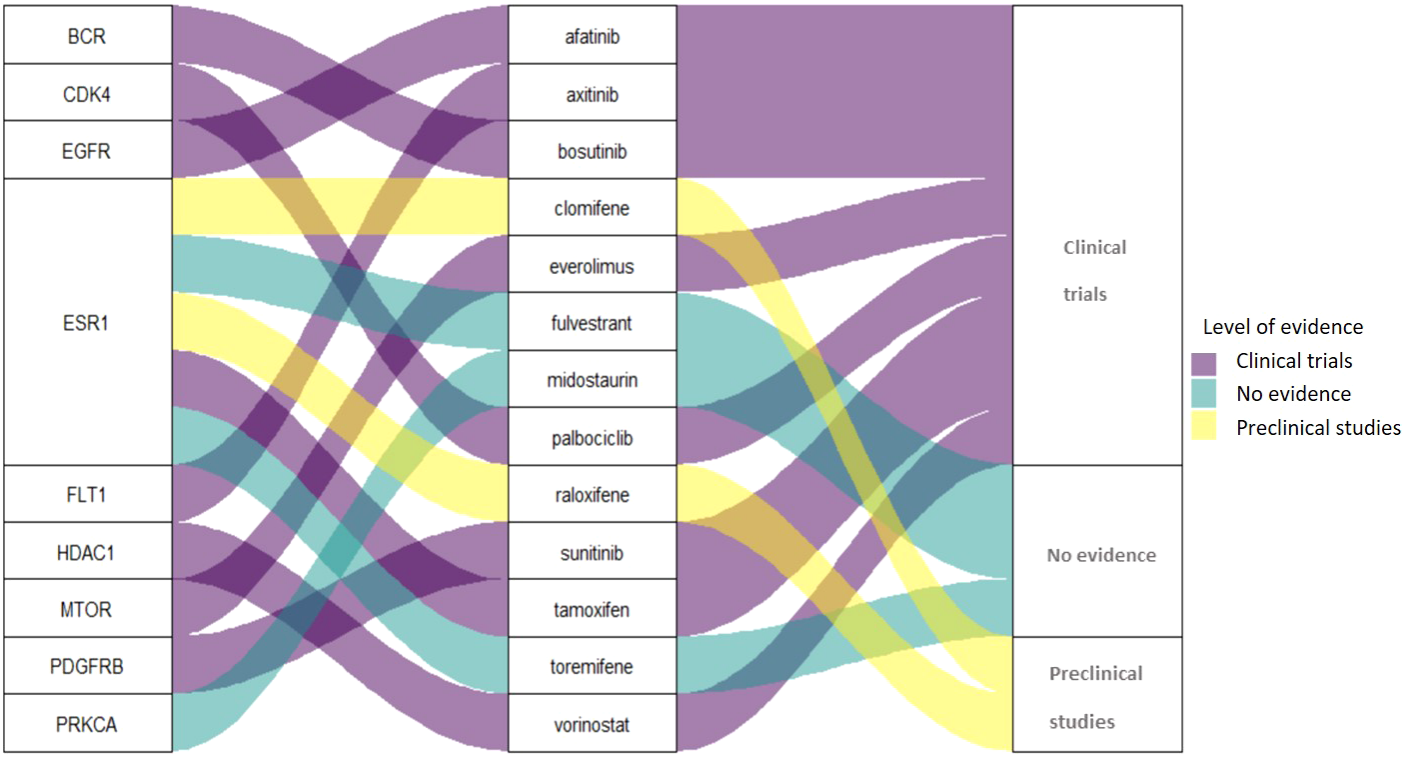
Connections among protein, candidate drugs, and evidence level for PC drug repurposing.

Among the 13 drugs extracted from the CMap Touchstone database, 8 drugs are under clinical trials for PC including tamoxifen, sunitinib, bosutinib, everolimus, afatinib, palbociclib, vorinostat, and axitinib. Two drugs are with preclinical studies clomifene and raloxifene; and three drugs are without evidence from clinical trials: fulvestrant, midostaurin, and toremifene. The connection among target proteins, PC candidate drugs, and the evidence level status is demonstrated in **Figure 4**. Among the 13 drugs, fulvestrant is a promising drug repurposing for PC because the target gene, ESR1, is one of the most dominated biological-PC risk genes in this analysis.

## Discussion

This study utilized a genomic database to retrieve somatic mutation genes of PC. Five functional annotations were applied to build a scoring system and prioritized PC risk drug target identifications. We found 318 drug target genes.12 genes of them had the highest score (score = 5) after prioritization. Molecular mechanism of the 12 genes were listed in **Table 3**. After prioritization of candidate drugs, we found 13 potential drugs (tamoxifen, sunitinib, bosutinib, everolimus, afatinib, palbociclib, vorinostat, axitinib, clomifene, raloxifene fulvestrant, midostaurin and toremifene) for PC.

**Table 3:** The molecular evidence of the 12 highest five functional annotations to PC.

Genes	Molecular mechanism	Sources
CDKN2A	Encodes p16 that recognizes tumor suppressor preventing progression through the G1 cell cycle checkpoint.	(27–29)
CTNNB1	Encodes β-catenin that is essential in the maintenance of epithelial cell layers and cell signaling.	(30–33)
BRCA2	An important player in the homologous DNA repair (HR) pathway and mutations.	(34–37)
AR	Androgen receptor (AR) signaling has an important role in the initiation and progression of many hormone-related cancers.	(38, 39)
CCND2	Cyclin D2 is a core component of the machinery that drives cell cycle progression.	(40)
PML	Involved in the regulation of cellular processes that are relevant to tumor suppression, such as DNA repair and the DNA damage response.	(41, 42)
MYC	C-Myc enhances aerobic glycolysis in cancer cells and regulates glutamate biosynthesis from glutamine.	(43, 44)
CCND3	CCND3 is the primary driver of the cell cycle that integrates extracellular mitogenic signaling.	(45, 46)
STAT3	STAT3 is a key element in multiple signaling pathways by promoting tumor progression, survival, tumor invasion, angiogenesis, and immunosuppression.	(47–49)
ZBTB16	ZBTB16 represses transcription and is associated with tumor progression.	(50)
EGFR	EGFR is a receptor tyrosine kinase of the ERB-B family that is abnormally activated in many epithelial tumors.	(24, 51, 52)
ESR1	The human estrogen receptor α (ERα), encoded by ESR1, is a member of the steroid/nuclear receptor superfamily and functions as a ligand-activated transcription factor.	(53, 54)

Out of 12 genes, some potential gene targets were highlighted by our pipelines, including BCR and MTOR (55, 56), which were targeted by bosutinib and everolimus, respectively. Genes including KRAS, TP53, and SMAD4 had also been reported from previous studies (57, 58). These results indicated that five functional annotations are useful tools to priortize the important genes.

CMap Touchstone analysis showed that ESR1 as a dominated target protein for PC. ESR1 is a ligand-activated transcription factor that belongs to the steroid/nuclear receptor superfamily (59). The estrogen-responsive element (ERE) on the promoters of ESR1 target genes dimerizes and binds to coactivators in response to estrogen binding (60). ESR1 is known to involve in various cancers, such as endometrial and ovarian cancers (61). A previous study mentioned that ESR1 is also expressed in a subset of pancreatic adenocarcinoma, most notably in mucinous tumors (55, 56). Tamoxifen is the ESR1-targeted drug with both preclinical trial and clinical results for PC (62, 63). Furthermore, several clinical trials have reported using estrogen therapy on PC treatment (53, 63–65). However, the results are still controversial. Another drug that targeted ESR1 is fulvestrant, originally used for metastatic breast cancer. Fulvestrant works by inhibiting the dimerization of the ESR1 receptor and exerts no estrogen agonist effect (66). Therefore, if compared with tamoxifen, side effects of fulvestrant seem to be more favorable.

Midostaurin is a candidate drug from our prioritization process. The target for midostaurin is PRKCA that is a phospholipid-dependent, cytoplasmic serine/threonine kinase acting as an intracellular signal transducer (67). PRKCA activates different proteins such as cellular proliferation, differentiation, and gene expression (68). In addition, the expression of PRKCA has been associated with an elevated expression of the multidrug resistance phenotype (MDR) (69). Clinically, PRKCA is a target for types of cancer including acute myeloid leukemia (AML), breast cancer, and ovarian cancer (70). However, the reference of PRKCA as a treatment target for PC is still limited. Therefore, we propose PRKCA as a potential novel target. In this study, midostaurin targeted PRKCA with a score of 89, indicating the similar profiles between midostaurin andgemcitabine.

This study focused on bioinformatic approaches to prioritize drug candidates for PC. However, we should emphasize that further experimental and clinical studies are necessary to confirm the findings. The advantage of these approaches is narrowing down the drug targets and improving the success rate of drug development by prioritizing the best candidates. It is worth noting that this study has some limitations. First, not all genes retrieved from our analysis were druggable. Second, we used only one drug, gemcitabine, as the standard treatment for PC; this approach omits other potential candidate drugs to be found. Finally, CMap did not provide specific cell lines for PC.

## Conclusions

Using our pipeline, we reported that there are 13 compounds that are the most potential PC candidate drugs. Two drugs are with preclinical results, eight are under clinical trials, and three have no previous reports yet. Two drugs (midostaurin and fulvestrant) were proposed as novel drugs repurposing for PC treatment. Here, we highlighted that our research demonstrated the viability of using public genomic information as a potential drug discovery method.

## Data availability statement

The original contributions presented in the study are included in the article/**Supplementary Material**. Further inquiries can be directed to the corresponding authors.

## Author contributions

Conceptualization: EM and W-CC. Data curation: EM, WA, LMI, C-NK and W-CC. Formal analyses: EM, WA, LMI and W-CC. Data interpretation and discussion: EM, WA, LMI, W-CH, C-NK and W-CC. Writing—review and editing: EM, WA, LMI, W-CH, C-NK and W-CC. Manuscript revision: EM, C-NK and W-CC. Supervision: W-CH, C-NK and W-CC. All authors contributed to the article and approved the submitted version.

## Funding

This work was supported by grants from the Ministry of Science and Technology, Taiwan (no. MOST109-2320-B-038-013) and Taipei Medical University, Taiwan (12310-106079;12310-10739) for Yusuke Nakamura Chair Professorship.

## Acknowledgments

We thank Min-Rou Lin, Wan-Hsuan Chou, Dr. Che-Mai Chang (Taipei Medical University, Taiwan) for helpful comments during the revision and Nien-Yu Yang (Taipei Medical University, Taiwan; The University of Manchester, UK) for the assistance with **Figure 1** draft.

## Conflict of interest

The authors declare that the research was conducted in the absence of any commercial or financial relationships that could be construed as a potential conflict of interest.

## Publisher’s note

All claims expressed in this article are solely those of the authors and do not necessarily represent those of their affiliated organizations, or those of the publisher, the editors and the reviewers. Any product that may be evaluated in this article, or claim that may be made by its manufacturer, is not guaranteed or endorsed by the publisher.

## Abbreviations

PC, pancreatic cancer; cBioPortal, Cancer genomic bio portal; MCF7, Michigan Cancer Foundation-7; RRM1, ribonucleoside-diphosphate reductase large subunit 1; PRKA, protein kinase A; ESR1, estrogen receptor 1; NCCN, National Comprehensive Cancer Network; ASCO, American Society of Clinical Oncology; KEGG, Kyoto Encyclopedia of Genes and Genomes; BP, biological process; KO, knockout mouse; GLAD4U, Gene List Automatically Derived For You; GSE, Gene Omnibus Dataset Series; ORA, Overrepresentation analysis; FDR, false discovery rate; CDKN2A, cyclin-dependent kinase inhibitor 2A; BRCA2, BReast CAncer gene 2; EGFR, epidermal growth factor receptor; BCR, breakpoint cluster region; MTOR, mammalian target of rapamycin; KRAS, K-Ras; TP53, tumor protein 53; SMAD4, suppressor of mothers against decapentaplegic 4; ERE, estrogen-responsive element; PRKCA, protein kinase C alpha; MDR, multidrug resistance phenotype; CML, chronic myelogenous leukemia; NSCLC, non-small cell lung cancer; PDGFRB, platelet-derived growth factor receptor beta; CDK4, cyclin-dependent kinase 4; FLT, fms-related receptor tyrosine kinase 1; HDAC1, histone deacetylase 1; CTNNB1, catenin beta 1; AR, androgen receptor; CCND2, cyclin D2; MYC, C-Myc; and CCND3, cyclin D3.

## Glossary

**Table:**

PC	pancreatic cancer
cBioPortal	Cancer genomic bio portal
MCF7	Michigan Cancer Foundation-7
RRM1	ribonucleoside-diphosphate, reductase large subunit 1
PRKA	protein kinase A
ESR1	estrogen receptor 1
NCCN	National Comprehensive Cancer Network
ASCO	American Society of Clinical Oncology
KEGG	Kyoto Encyclopedia of Genes and Genomes
BP	biological process
KO	knockout mouse
GLAD4U	Gene List Automatically Derived For You
GSE	Gene Omnibus Dataset Series
ORA	Overrepresentation analysis
FDR	false discovery rate
CDKN2A	Cyclindependent kinase inhibitor 2A
BRCA2	BReast CAncer gene 2
EGFR	epidermal growth factor receptor
BCR	breakpoint cluster region
MTOR	mammalian target of rapamycin
KRAS	K-Ras
TP53	tumor protein 53
SMAD4	suppressor of mothers against decapentaplegic 4
ERE	estrogenresponsive element
PRKCA	protein kinase C alpha
MDR	multidrug resistance phenotype
CML	chronic myelogenous leukemia
NSCLC	nonsmall cell lung cancer
PDGFRB	platelet-derived growth factor receptor beta
CDK4	cyclin-dependent kinase 4
FLT	fms-related receptor tyrosine kinase 1
HDAC1	histone deacetylase 1
CTNNB1	catenin beta 1
AR	androgen receptor
CCND2	cyclin D2
MYC	C-Myc
CCND3	cyclin D3
GEO	Gene Expression Omnibus
