# Supplementary material for: Integrated genomic analysis to identify druggable targets for pancreatic cancer
Source: Front Oncol. 2022 Dec 1;12:989077. doi: 10.3389/fonc.2022.989077 (PMC9752886; doi:10.3389/fonc.2022.989077)
Supplement: Supplementary file 1 [file DataSheet_1.docx]

Supplementary

| 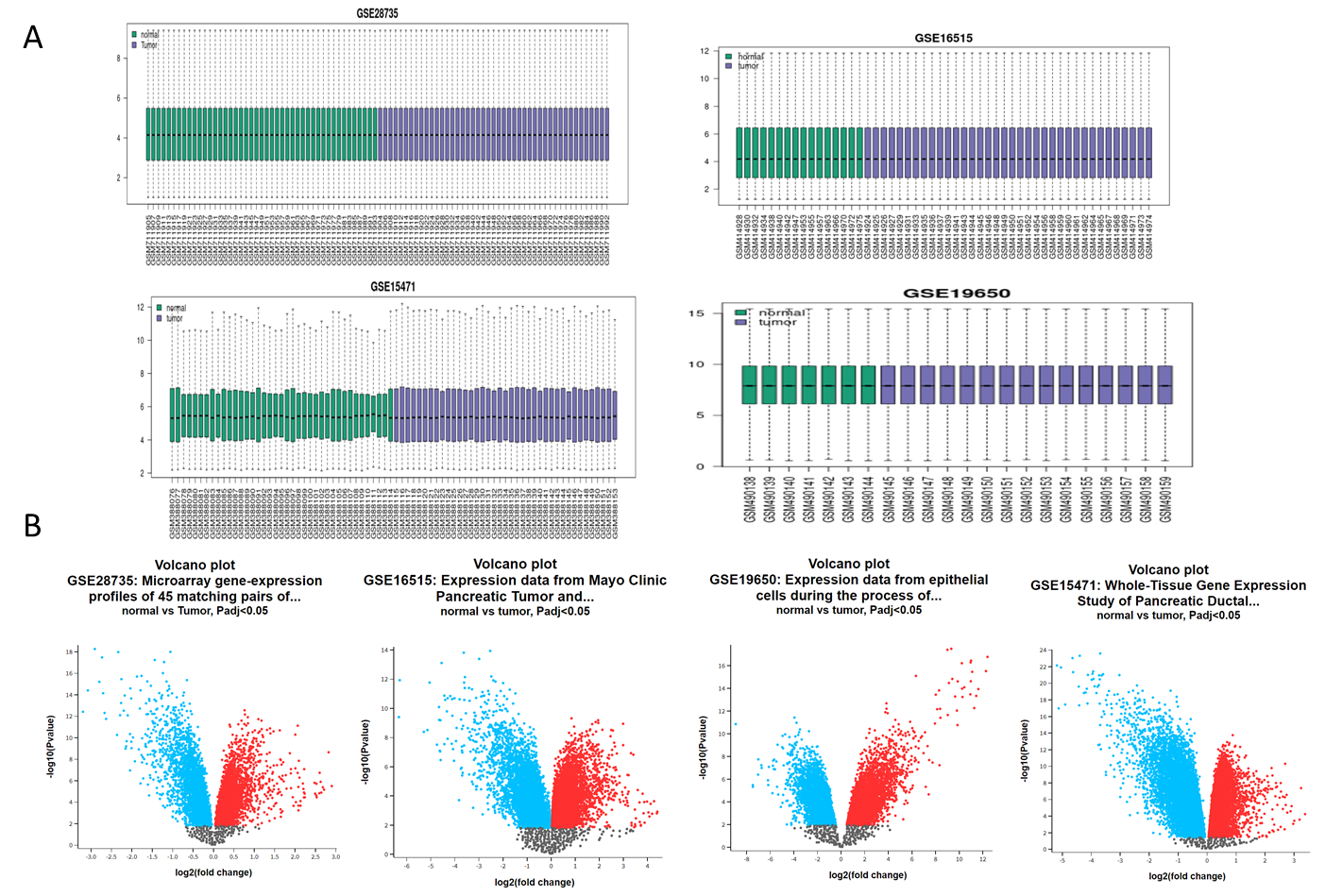 |
| --- |
| **Supplementary Figure 1**. Applying normalization for the technical and biological variations from 4 GEO datasets (GSSE28735, GSE15471, GSE16515, and GSE 19650). (A) Normalized distribution and (B) volcano plot four datasets. Differential expression gene was retrieved with criteria adjusted p value cutoff of 0.05. |


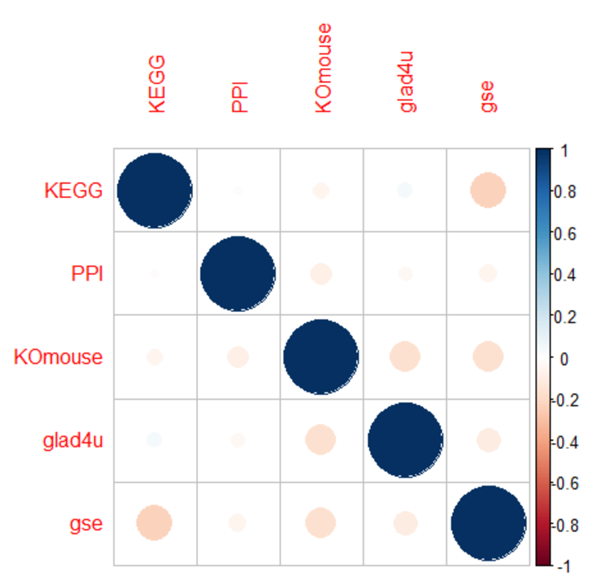


**Supplementary Figure 2.** A Phi correlation was utilized to determine whether each functional annotation is an independent parameter. The pairwise Phi correlation coefficient between the five criteria is represented via a correlogram. A positive correlation is indicated by the blue color, whereas a negative correlation is indicated by the red color.

**Supplementary Table 1.** Nine studies related to pancreatic cancer that retrieved from cBioportal

| No | Studies | Sample numbers |
| --- | --- | --- |
| 1  2  3  4  5  6  7  8  9 | Acinar Cell Carcinoma of the Pancreas (JHU, J Pathol 2014)  Cystic Tumor of the Pancreas (Johns Hopkins, PNAS 2011)  Pancreatic Cancer (Shanghai, Nat Commun 2013)  Pancreatic Adenocarcinoma (ICGC, Nature 2012)  Pancreatic Adenocarcinoma (QCMG, Nature 2016)  Pancreatic Adenocarcinoma (TCGA, Firehose Legacy)  Pancreatic Cancer (UTSW, Nat Commun 2015)  Pancreatic Neuroendocrine Tumors (Johns Hopkins University, Science 2011)  Pancreatic Neuroendocrine Tumors (Multi-Institute, Nature 2017) | 23  32  10  99  456  186  109  32  98 |

**Supplementary Table 2.** PC-associated genes

| *CDKN2A*  *CTNNB1*  *BRCA2*  *AR*  *CCND2*  *PML*  *MYC*  *CCND3*  *STAT3*  *ZBTB16*  *EGFR*  *ESR1*  *TGFBR1*  *MECOM*  *HGF*  *FGFR1*  *PDGFRA*  *FLT3*  *AKT3*  *KRAS*  *SMAD4*  *APC*  *PTEN*  *BRAF*  *MTOR*  *RB1*  *BRCA1*  *MST1R*  *NOTCH1*  *NOTCH3*  *KLF5*  *KIT*  *PTCH1*  *TERT*  *KLF4*  *SOX17*  *ALK* | *MET*  *MITF*  *PPARG*  *ABL1*  *AKT1*  *XIAP*  *STAT5A*  *STAT5B*  *TCF7L2*  *VEGFA*  *PAX8*  *BAX*  *BCL2L1*  *CCNE1*  *CDK4*  *ETS1*  *HRAS*  *IGF2*  *NFIB*  *NRAS*  *RARA*  *SFRP1*  *LEF1*  *TP53*  *TGFBR2*  *NTRK3*  *FLT4*  *MKI67*  *ERBB4*  *RUNX1T1*  *TP63*  *ERBB2*  *IGF1R*  *SMO*  *GLI1*  *HIF1A*  *VHL* | *E2F3*  *ERG*  *FGFR3*  *GSK3B*  *IGF1*  *KDR*  *PDGFB*  *PIM1*  *SFRP4*  *BTG2*  *VTCN1*  *ZNF703*  *CDKN2B*  *FOXO1*  *JAK2*  *SOS1*  *MEN1*  *GNAS*  *ATM*  *NF1*  *PIK3CA*  *NOTCH2*  *PGR*  *LRRK2*  *SMAD3*  *RET*  *INHBA*  *LRP5*  *ERBB3*  *NRG1*  *IL7R*  *MSH2*  *DDR2*  *BCL11B*  *NBN*  *NF2*  *NTRK2* | *CSF1R*  *CYP19A1*  *ETV5*  *ETV6*  *SOX9*  *WT1*  *CXCR4*  *NCOA3*  *BAP1*  *TET1*  *PRDM1*  *CTLA4*  *EGR1*  *ERCC2*  *FOXO3*  *GATA2*  *GATA3*  *PIK3CB*  *BRIP1*  *BCL6*  *FCGR2B*  *IL6ST*  *JUN*  *LIFR*  *NFKBIA*  *PLAG1*  *PRKAR1A*  *SIX1*  *SOX2*  *STAT1*  *SYK*  *CDKN2C*  *CRKL*  *FGF10*  *FLI1*  *MLH1*  *RXRA* | *PREX2*  *CDH11*  *STK11*  *SETBP1*  *FBXW7*  *AXL*  *ARHGEF12*  *BARD1*  *FGF6*  *NTRK1*  *ROS1*  *SMARCB1*  *DICER1*  *FLT1*  *HDAC1*  *LRP6*  *PLCG1*  *PRKD1*  *STIL*  *EML4*  *SALL4*  *PALB2*  *CASP8*  *CTNNA1*  *DNMT1*  *EPAS1*  *SMAD2*  *MSH3*  *REST*  *BCR*  *CALR*  *GNB1*  *MSH6*  *IDH2*  *PRRX1*  *PRKCI*  *MAPK1* | *CD276*  *FLCN*  *BUB1B*  *CBFA2T3*  *CDH1*  *FGF3*  *FH*  *GPC3*  *NFE2L2*  *MAP2K1*  *RAC2*  *INPP4B*  *TFG*  *DKK3*  *CD274*  *RHOA*  *ELF4*  *EZH2*  *FGF4*  *MAX*  *PTK6*  *TAL1*  *NCOA4*  *YAP1*  *SUZ12*  *EGFL7*  *ACKR3*  *ACVR2A*  *RNF213*  *CREBBP*  *JAK1*  *PTPRS*  *EP300*  *FANCD2*  *PIK3CG*  *PRKDC*  *ROBO1* | *TET2*  *CYLD*  *DNMT3B*  *STAT4*  *NKX2-1*  *NCOA1*  *DTX1*  *FANCA*  *FOXA1*  *NFATC2*  *PIK3CD*  *PMS2*  *PTPN6*  *CRTC1*  *RUNX2*  *CBL*  *INPP5D*  *INSR*  *JARID2*  *NFKB2*  *MERTK*  *SETD5*  *ASXL1*  *CSF1*  *CSF3R*  *HOXA3*  *IFNGR1*  *PAX3*  *PBX1*  *PDCD1*  *EZR*  *FGF23*  *SPOP*  *SOCS2*  *CARM1*  *ARAF*  *ETV4* | *TSHR*  *RECQL4*  *HOXB13*  *ARID5B*  *MSI2*  *DDR1*  *CD36*  *INHA*  *LMNA*  *PDK1*  *PTPN2*  *RAD51C*  *TRAF3*  *TNFRSF11A*  *FSTL3*  *BRD4*  *DAZAP1*  *ARID1A*  *FAT4*  *KMT2D*  *RBM10*  *SETD2*  *RELN*  *ARID2*  *ZNF521*  *MYH11*  *POLE*  *PTPRD*  *EPHA7*  *KMT2A*  *PLCG2*  *TPR*  *AXIN1*  *MED12*  *KNL1*  *MEF2C*  *ADGRA2* | *AKT2*  *CDK6*  *CDKN1A*  *FGFR2*  *JAK3*  *MDM2*  *AXIN2*  *ARNT*  *FGFR4*  *SFRP2*  *WWTR1*  *FOXP1*  *PDGFRB*  *PTPRC*  *STAT6*  *TBX3*  *IKZF1*  *CDC73*  *PPM1D*  *BCL2L11*  *NDRG1*  *RNF43*  *PBRM1*  *TSC2*  *RAF1*  *SRC*  *TFE3*  *TSC1*  *MALT1*  *KDM4C*  *COL1A1*  *IRS2*  *LATS1*  *IRS1*  *PAX7*  *BCL11A*  *EWSR1* | *FES*  *HOXD13*  *HSP90AA1*  *IRF1*  *TRAF2*  *UBR5*  *RICTOR*  *DNMT3A*  *NOTCH4*  *CIC*  *RPS6KB2*  *SSX1*  *ELOC*  *TPM3*  *TRAF5*  *CNBP*  *PICALM*  *TRIM24*  *PHOX2B*  *HIST1H2BJ*  *PCSK7*  *AURKB*  *TCL1B*  *FRS2*  *WIF1*  *NCSTN*  *YPEL5*  *DUSP22*  *PGBD5*  *NUF2*  *SLC45A3*  *TNFRSF17*  *BLM*  *BTK*  *CDC42*  *CHN1*  *CYP17A1* | *USP8*  *HDAC4*  *DDX41*  *KDM2B*  *CCNB3*  *PARP1*  *EPS15*  *EXT2*  *FGF12*  *INPPL1*  *MEF2D*  *MSI1*  *MYCN*  *FURIN*  *TLE1*  *ZNF217*  *MLLT10*  *CTCF*  *CHEK2*  *SAMHD1*  *LATS2*  *CARD11*  *DOT1L*  *ALB*  *BMPR1A*  *ZFP36L1*  *CD70*  *EPHB4*  *ERCC5*  *EXT1*  *EIF4E*  *ELN*  *EPHA3*  *ETV1*  *H3F3A*  *HLF*  *TLX1* |
| --- | --- | --- | --- | --- | --- | --- | --- | --- | --- | --- |

| *GNAQ*  *IKBKB*  *IRF2*  *MN1*  *MSN*  *MUTYH*  *MAP2K2*  *SGK1*  *SS18*  *STAT2*  *TNFAIP3*  *CCDC6*  *SMC1A*  *HIST1H2AC*  *CUL3*  *MAGED1*  *KEAP1*  *GAB2*  *ARHGAP26*  *ACSL6*  *ARID4B*  *PAK5*  *SMYD3*  *RBM15*  *ZNRF3*  *MAML2*  *AMER1*  *NEGR1*  *CD79A*  *KLF6*  *DDB2*  *DNM2*  *EIF4A2*  *EPOR*  *ERCC4*  *FHIT* | *FOXF1*  *GATA1*  *GNA11*  *KCNJ5*  *LMO2*  *LTK*  *MTAP*  *PIK3R1*  *PIK3R2*  *PRKACA*  *PTK7*  *HOXA9*  *HSP90AB1*  *IDH1*  *KLK2*  *MLLT3*  *MRE11*  *MTCP1*  *OMD*  *PAX5*  *POU5F1*  *RALGDS*  *TRIM27*  *SDC4*  *SDHC*  *SLC1A2*  *AURKA*  *TFRC*  *NSD2*  *XPC*  *NR4A3*  *PIK3R3*  *TNFRSF14*  *S1PR2*  *QKI*  *ABI1*  *FGFR1OP* | *TLX3*  *ZBTB7A*  *GTSE1*  *SUFU*  *SETD4*  *YY1AP1*  *HOOK3*  *RSPO3*  *MPEG1*  *NUTM1*  *ACVR1B*  *SMARCA4*  *CHD2*  *PRSS1*  *ATR*  *CACNA1D*  *POLD1*  *TLE4*  *NCOA2*  *CUX1*  *ARID4A*  *NCOR1*  *SPEN*  *ITK*  *WRN*  *PASK*  *DDX4*  *ACTB*  *HNF1A*  *VAV1*  *WAS*  *FANCF*  *GATA4*  *GTF2I*  *HIP1*  *AFF1*  *PRF1* | *TLE3*  *KAT6A*  *MDC1*  *PSIP1*  *PATZ1*  *AFF4*  *IL21R*  *PRDM16*  *SLX4*  *RHOH*  *ELK4*  *FUS*  *LCK*  *CIITA*  *PAFAH1B2*  *RPL22*  *TCF12*  *KMT2B*  *RAD50*  *TIPARP*  *TENT5C*  *PAG1*  *CCNB1IP1*  *RSPO2*  *ATP6V1B2*  *FANCC*  *FANCG*  *HOXA13*  *PPP2R2A*  *PTPN1*  *TCF3*  *VAV2*  *SOCS1*  *FUBP1*  *BCL10*  *ATG5*  *MAD2L2* | *CRBN*  *MRTFA*  *COP1*  *SETD3*  *LRP1B*  *KMT2C*  *DAXX*  *MAP2K4*  *KDM6A*  *PCLO*  *ATRX*  *FAT1*  *PTPRT*  *SF3B1*  *KSR2*  *ZFHX3*  *TAF1*  *GRIN2A*  *ARHGAP35*  *U2AF1*  *EP400*  *LZTR1*  *SNCAIP*  *CHD4*  *COL2A1*  *EPHA5*  *MYH9*  *NCOR2*  *GRM3*  *NUP98*  *CLIP1*  *TP53BP1*  *KDM5C*  *TRRAP*  *PDE4DIP*  *WDR90*  *AFF3* | *NUMA1*  *RANBP2*  *CLTCL1*  *IRS4*  *GAS7*  *TRIP11*  *SETD1A*  *BCORL1*  *ARHGEF28*  *DDX3X*  *DDX10*  *KEL*  *MYO5A*  *PPP2R1A*  *PTPN13*  *PTPRB*  *SMARCA1*  *TYK2*  *MGAM*  *USP6*  *ZMYM3*  *CTR9*  *POLQ*  *SEC31A*  *PDCD11*  *SMG1*  *SETD1B*  *ANKRD11*  *NIN*  *TRIM33*  *BCOR*  *WDCP*  *BRSK1*  *SNX29*  *LRIG3*  *MYO18A*  *CAD* | *EBF1*  *ELF3*  *HIST1H1E*  *LPP*  *CNOT3*  *PER1*  *PIK3C2B*  *RAD21*  *TAP1*  *TLE2*  *XPO1*  *YY1*  *GMPS*  *SETDB1*  *FAF1*  *DKK2*  *CDK12*  *NSD3*  *EMSY*  *MIB1*  *ZNF750*  *FIP1L1*  *FCRL4*  *MBD6*  *ARID3C*  *BCL9*  *FANCE*  *HLA-A*  *KTN1*  *LCP1*  *MLLT1*  *AFDN*  *PRKN*  *PC*  *PHF1*  *PIK3C3*  *PRCC* | *PTPRO*  *RASA1*  *KDM5A*  *SERPINB4*  *SMARCE1*  *CSDE1*  *NUP214*  *HIST1H2BO*  *MAP3K6*  *STAG2*  *SP140*  *IKZF2*  *ERC1*  *MGA*  *RYBP*  *POT1*  *ZBTB20*  *SND1*  *DROSHA*  *HDAC7*  *RTEL1*  *ELP2*  *ASXL2*  *CMTR2*  *PRDM14*  *NSD1*  *SPRTN*  *TRAF7*  *TET3*  *ATIC*  *ATP2B3*  *CBLB*  *CPS1*  *DCTN1*  *ARID3A*  *ERCC3*  *EZH1* | *ACSL3*  *DNAJB1*  *MAP3K1*  *NFKBIE*  *PIGA*  *PIK3C2G*  *PMS1*  *RECQL*  *RIT1*  *ATXN7*  *SDHA*  *SMARCD1*  *MAP3K7*  *TOP1*  *HIRA*  *YWHAE*  *ZMYM2*  *BRD3*  *HIST3H3*  *RAD54L*  *PPFIBP1*  *EED*  *HERPUD1*  *SH2B3*  *STAG1*  *SLC34A2*  *SEPTIN9*  *U2AF2*  *RRAS2*  *DIS3*  *NT5C2*  *LARP4B*  *CAMTA1*  *KAT6B*  *DCUN1D1*  *SHQ1*  *KBTBD4* | *ARID1B*  *CREB3L2*  *SETD6*  *SESN2*  *FLYWCH1*  *SPRED1*  *RASGEF1A*  *P2RY8*  *ACTG1*  *ATF1*  *ATP1A1*  *ATP6AP1*  *BCL3*  *BCL7A*  *CD58*  *CD79B*  *CDK8*  *CLTC*  *DDX6*  *DUSP2*  *GABRA6*  *HIST1H1C*  *HIST1H1D*  *H3F3B*  *HLA-B*  *HOXC11*  *HOXC13*  *HOXD11*  *IRF8*  *INPP4A*  *MLF1*  *NACA*  *NONO*  *PCBP1*  *UPF1*  *RPL10*  *RRAS* | *SH3GL1*  *TAP2*  *TCEA1*  *ZNF24*  *ELL*  *HIST1H2BG*  *HIST1H3I*  *RPS6KA4*  *SMC3*  *APOBEC3B*  *NUP93*  *GOLGA5*  *PARP2*  *GPHN*  *NOD1*  *ARID3B*  *CCT6B*  *PLK2*  *ARID5A*  *CLP1*  *CNTRL*  *ICK* |
| --- | --- | --- | --- | --- | --- | --- | --- | --- | --- | --- |
| *FNBP1*  *DKK4*  *AGO2*  *ETAA1*  *TMEM30A*  *NADK*  *ASPSCR1*  *CHCHD7*  *FBXO31*  *FBXO11*  *STK40*  *PHF6*  *KLHL6*  *CCNQ*  *SESN3*  *BTLA*  *PPP4R2*  *ZNF384*  *BCL9L*  *ECT2L*  *ACVR1*  *CARS*  *DDX5*  *DUSP4*  *DUSP9*  *EIF1AX*  *ERF*  *KDSR*  *HIST1H2BD*  *HNRNPA2B1*  *HSD3B1*  *EIF3E*  *KIF5B*  *SH2D1A*  *MAF* | *MLLT6*  *NFE2*  *SEPTIN5*  *POU2AF1*  *PPP1CB*  *PPP6C*  *RAD52*  *RAP1GDS1*  *REL*  *RPN1*  *SET*  *SFPQ*  *SRSF2*  *SRSF3*  *TAL2*  *TYRO3*  *YES1*  *PTP4A1*  *SHOC2*  *TAF15*  *ZRSR2*  *HIST1H2AM*  *HIST1H3G*  *HIST1H3J*  *MKNK1*  *CCN6*  *RABEP1*  *IKBKE*  *ARFRP1*  *MLLT11*  *ICOSLG*  *SESN1*  *APH1A*  *CYSLTR2*  *BACH2*  *RRAGC*  *TBL1XR1* | *SETD7*  *SETDB2*  *ABRAXAS1*  *AJUBA*  *CREB3L1*  *JAZF1*  *KMT5A*  *SERP2* |  |  |  |  |  |  |  |  |

**SupplementaryTable 3.** Biological-PC risk genes

| **Gene code symbol** | **Biological risk gene of PC** | | | | | **Score** |
| --- | --- | --- | --- | --- | --- | --- |
|  | **KEGG** | **BP** | **KO Mouse** | **GLAD4U** | **GSE** |  |
| *CDKN2A* |  |  |  |  |  | 5 |
| *CTNNB1* |  |  |  |  |  | 5 |
| *BRCA2* |  |  |  |  |  | 5 |
| *AR* |  |  |  |  |  | 5 |
| *CCND2* |  |  |  |  |  | 5 |
| *PML* |  |  |  |  |  | 5 |
| *MYC* |  |  |  |  |  | 5 |
| *CCND3* |  |  |  |  |  | 5 |
| *STAT3* |  |  |  |  |  | 5 |
| *ZBTB16* |  |  |  |  |  | 5 |
| *EGFR* |  |  |  |  |  | 5 |
| *ESR1* |  |  |  |  |  | 5 |
| *TGFBR1* |  |  |  |  |  | 4 |
| *MECOM* |  |  |  |  |  | 4 |
| *HGF* |  |  |  |  |  | 4 |
| *FGFR1* |  |  |  |  |  | 4 |
| *PDGFRA* |  |  |  |  |  | 4 |
| *FLT3* |  |  |  |  |  | 4 |
| *AKT3* |  |  |  |  |  | 4 |
| *KRAS* |  |  |  |  |  | 4 |
| *SMAD4* |  |  |  |  |  | 4 |
| *APC* |  |  |  |  |  | 4 |
| *PTEN* |  |  |  |  |  | 4 |
| *BRAF* |  |  |  |  |  | 4 |
| *MTOR* |  |  |  |  |  | 4 |
| *RB1* |  |  |  |  |  | 4 |
| *BRCA1* |  |  |  |  |  | 4 |
| *MST1R* |  |  |  |  |  | 4 |
| *NOTCH1* |  |  |  |  |  | 4 |
| *NOTCH3* |  |  |  |  |  | 4 |
| *KLF5* |  |  |  |  |  | 4 |
| *KIT* |  |  |  |  |  | 4 |
| *PTCH1* |  |  |  |  |  | 4 |
| *TERT* |  |  |  |  |  | 4 |
| *KLF4* |  |  |  |  |  | 4 |
| *SOX17* |  |  |  |  |  | 4 |
| *ALK* |  |  |  |  |  | 4 |
| *AKT2* |  |  |  |  |  | 4 |
| *CDK6* |  |  |  |  |  | 4 |
| *CDKN1A* |  |  |  |  |  | 4 |
| *FGFR2* |  |  |  |  |  | 4 |
| *JAK3* |  |  |  |  |  | 4 |
| *MDM2* |  |  |  |  |  | 4 |
| *MET* |  |  |  |  |  | 4 |
| *MITF* |  |  |  |  |  | 4 |
| *PPARG* |  |  |  |  |  | 4 |
| *ABL1* |  |  |  |  |  | 4 |
| *AKT1* |  |  |  |  |  | 4 |
| *XIAP* |  |  |  |  |  | 4 |
| *STAT5A* |  |  |  |  |  | 4 |
| *STAT5B* |  |  |  |  |  | 4 |
| *TCF7L2* |  |  |  |  |  | 4 |
| *VEGFA* |  |  |  |  |  | 4 |
| *PAX8* |  |  |  |  |  | 4 |
| *BAX* |  |  |  |  |  | 4 |
| *BCL2L1* |  |  |  |  |  | 4 |
| *CCNE1* |  |  |  |  |  | 4 |
| *CDK4* |  |  |  |  |  | 4 |
| *ETS1* |  |  |  |  |  | 4 |
| *HRAS* |  |  |  |  |  | 4 |
| *IGF2* |  |  |  |  |  | 4 |
| *NFIB* |  |  |  |  |  | 4 |
| *NRAS* |  |  |  |  |  | 4 |
| *RARA* |  |  |  |  |  | 4 |
| *SFRP1* |  |  |  |  |  | 4 |
| *LEF1* |  |  |  |  |  | 4 |
| *TP53* |  |  |  |  |  | 3 |
| *TGFBR2* |  |  |  |  |  | 3 |
| *NTRK3* |  |  |  |  |  | 3 |
| *FLT4* |  |  |  |  |  | 3 |
| *MKI67* |  |  |  |  |  | 3 |
| *ERBB4* |  |  |  |  |  | 3 |
| *RUNX1T1* |  |  |  |  |  | 3 |
| *TP63* |  |  |  |  |  | 3 |
| *ERBB2* |  |  |  |  |  | 3 |
| *IGF1R* |  |  |  |  |  | 3 |
| *SMO* |  |  |  |  |  | 3 |
| *GLI1* |  |  |  |  |  | 3 |
| *HIF1A* |  |  |  |  |  | 3 |
| *VHL* |  |  |  |  |  | 3 |
| *AXIN2* |  |  |  |  |  | 3 |
| *ARNT* |  |  |  |  |  | 3 |
| *FGFR4* |  |  |  |  |  | 3 |
| *SFRP2* |  |  |  |  |  | 3 |
| *WWTR1* |  |  |  |  |  | 3 |
| *FOXP1* |  |  |  |  |  | 3 |
| *E2F3* |  |  |  |  |  | 3 |
| *ERG* |  |  |  |  |  | 3 |
| *FGFR3* |  |  |  |  |  | 3 |
| *GSK3B* |  |  |  |  |  | 3 |
| *IGF1* |  |  |  |  |  | 3 |
| *KDR* |  |  |  |  |  | 3 |
| *PDGFB* |  |  |  |  |  | 3 |
| *PIM1* |  |  |  |  |  | 3 |
| *SFRP4* |  |  |  |  |  | 3 |
| *BTG2* |  |  |  |  |  | 3 |
| *VTCN1* |  |  |  |  |  | 3 |
| *ZNF703* |  |  |  |  |  | 3 |
| *CDKN2B* |  |  |  |  |  | 3 |
| *FOXO1* |  |  |  |  |  | 3 |
| *JAK2* |  |  |  |  |  | 3 |
| *SOS1* |  |  |  |  |  | 3 |
| *MEN1* |  |  |  |  |  | 3 |
| *GNAS* |  |  |  |  |  | 3 |
| *ATM* |  |  |  |  |  | 3 |
| *NF1* |  |  |  |  |  | 3 |
| *PIK3CA* |  |  |  |  |  | 3 |
| *NOTCH2* |  |  |  |  |  | 3 |
| *PGR* |  |  |  |  |  | 3 |
| *LRRK2* |  |  |  |  |  | 3 |
| *SMAD3* |  |  |  |  |  | 3 |
| *RET* |  |  |  |  |  | 3 |
| *INHBA* |  |  |  |  |  | 3 |
| *LRP5* |  |  |  |  |  | 3 |
| *ERBB3* |  |  |  |  |  | 3 |
| *NRG1* |  |  |  |  |  | 3 |
| *IL7R* |  |  |  |  |  | 3 |
| *MSH2* |  |  |  |  |  | 3 |
| *DDR2* |  |  |  |  |  | 3 |
| *BCL11B* |  |  |  |  |  | 3 |
| *NBN* |  |  |  |  |  | 3 |
| *NF2* |  |  |  |  |  | 3 |
| *NTRK2* |  |  |  |  |  | 3 |
| *PDGFRB* |  |  |  |  |  | 3 |
| *PTPRC* |  |  |  |  |  | 3 |
| *STAT6* |  |  |  |  |  | 3 |
| *TBX3* |  |  |  |  |  | 3 |
| *IKZF1* |  |  |  |  |  | 3 |
| *CDC73* |  |  |  |  |  | 3 |
| *CSF1R* |  |  |  |  |  | 3 |
| *CYP19A1* |  |  |  |  |  | 3 |
| *ETV5* |  |  |  |  |  | 3 |
| *ETV6* |  |  |  |  |  | 3 |
| *SOX9* |  |  |  |  |  | 3 |
| *WT1* |  |  |  |  |  | 3 |
| *CXCR4* |  |  |  |  |  | 3 |
| *NCOA3* |  |  |  |  |  | 3 |
| *BAP1* |  |  |  |  |  | 3 |
| *TET1* |  |  |  |  |  | 3 |
| *PRDM1* |  |  |  |  |  | 3 |
| *CTLA4* |  |  |  |  |  | 3 |
| *EGR1* |  |  |  |  |  | 3 |
| *ERCC2* |  |  |  |  |  | 3 |
| *FOXO3* |  |  |  |  |  | 3 |
| *GATA2* |  |  |  |  |  | 3 |
| *GATA3* |  |  |  |  |  | 3 |
| *PIK3CB* |  |  |  |  |  | 3 |
| *BRIP1* |  |  |  |  |  | 3 |
| *BCL6* |  |  |  |  |  | 3 |
| *FCGR2B* |  |  |  |  |  | 3 |
| *IL6ST* |  |  |  |  |  | 3 |
| *JUN* |  |  |  |  |  | 3 |
| *LIFR* |  |  |  |  |  | 3 |
| *NFKBIA* |  |  |  |  |  | 3 |
| *PLAG1* |  |  |  |  |  | 3 |
| *PRKAR1A* |  |  |  |  |  | 3 |
| *SIX1* |  |  |  |  |  | 3 |
| *SOX2* |  |  |  |  |  | 3 |
| *STAT1* |  |  |  |  |  | 3 |
| *SYK* |  |  |  |  |  | 3 |
| *CDKN2C* |  |  |  |  |  | 3 |
| *CRKL* |  |  |  |  |  | 3 |
| *FGF10* |  |  |  |  |  | 3 |
| *FLI1* |  |  |  |  |  | 3 |
| *MLH1* |  |  |  |  |  | 3 |
| *RXRA* |  |  |  |  |  | 3 |
| *PPM1D* |  |  |  |  |  | 3 |
| *BCL2L11* |  |  |  |  |  | 3 |
| *NDRG1* |  |  |  |  |  | 3 |
| *RNF43* |  |  |  |  |  | 2 |
| *PBRM1* |  |  |  |  |  | 2 |
| *TSC2* |  |  |  |  |  | 2 |
| *PREX2* |  |  |  |  |  | 2 |
| *CDH11* |  |  |  |  |  | 2 |
| *STK11* |  |  |  |  |  | 2 |
| *SETBP1* |  |  |  |  |  | 2 |
| *FBXW7* |  |  |  |  |  | 2 |
| *AXL* |  |  |  |  |  | 2 |
| *ARHGEF12* |  |  |  |  |  | 2 |
| *BARD1* |  |  |  |  |  | 2 |
| *FGF6* |  |  |  |  |  | 2 |
| *NTRK1* |  |  |  |  |  | 2 |
| *ROS1* |  |  |  |  |  | 2 |
| *SMARCB1* |  |  |  |  |  | 2 |
| *DICER1* |  |  |  |  |  | 2 |
| *FLT1* |  |  |  |  |  | 2 |
| *HDAC1* |  |  |  |  |  | 2 |
| *LRP6* |  |  |  |  |  | 2 |
| *PLCG1* |  |  |  |  |  | 2 |
| *PRKD1* |  |  |  |  |  | 2 |
| *STIL* |  |  |  |  |  | 2 |
| *EML4* |  |  |  |  |  | 2 |
| *SALL4* |  |  |  |  |  | 2 |
| *PALB2* |  |  |  |  |  | 2 |
| *CASP8* |  |  |  |  |  | 2 |
| *CTNNA1* |  |  |  |  |  | 2 |
| *DNMT1* |  |  |  |  |  | 2 |
| *EPAS1* |  |  |  |  |  | 2 |
| *SMAD2* |  |  |  |  |  | 2 |
| *MSH3* |  |  |  |  |  | 2 |
| *REST* |  |  |  |  |  | 2 |
| *BCR* |  |  |  |  |  | 2 |
| *CALR* |  |  |  |  |  | 2 |
| *GNB1* |  |  |  |  |  | 2 |
| *MSH6* |  |  |  |  |  | 2 |
| *IDH2* |  |  |  |  |  | 2 |
| *PRRX1* |  |  |  |  |  | 2 |
| *PRKCI* |  |  |  |  |  | 2 |
| *MAPK1* |  |  |  |  |  | 2 |
| *RAF1* |  |  |  |  |  | 2 |
| *SRC* |  |  |  |  |  | 2 |
| *TFE3* |  |  |  |  |  | 2 |
| *TSC1* |  |  |  |  |  | 2 |
| *MALT1* |  |  |  |  |  | 2 |
| *KDM4C* |  |  |  |  |  | 2 |
| *CD276* |  |  |  |  |  | 2 |
| *FLCN* |  |  |  |  |  | 2 |
| *BUB1B* |  |  |  |  |  | 2 |
| *CBFA2T3* |  |  |  |  |  | 2 |
| *CDH1* |  |  |  |  |  | 2 |
| *FGF3* |  |  |  |  |  | 2 |
| *FH* |  |  |  |  |  | 2 |
| *GPC3* |  |  |  |  |  | 2 |
| *NFE2L2* |  |  |  |  |  | 2 |
| *MAP2K1* |  |  |  |  |  | 2 |
| *RAC2* |  |  |  |  |  | 2 |
| *INPP4B* |  |  |  |  |  | 2 |
| *TFG* |  |  |  |  |  | 2 |
| *DKK3* |  |  |  |  |  | 2 |
| *CD274* |  |  |  |  |  | 2 |
| *RHOA* |  |  |  |  |  | 2 |
| *ELF4* |  |  |  |  |  | 2 |
| *EZH2* |  |  |  |  |  | 2 |
| *FGF4* |  |  |  |  |  | 2 |
| *MAX* |  |  |  |  |  | 2 |
| *PTK6* |  |  |  |  |  | 2 |
| *TAL1* |  |  |  |  |  | 2 |
| *NCOA4* |  |  |  |  |  | 2 |
| *YAP1* |  |  |  |  |  | 2 |
| *SUZ12* |  |  |  |  |  | 2 |
| *EGFL7* |  |  |  |  |  | 2 |
| *ACKR3* |  |  |  |  |  | 2 |
| *ACVR2A* |  |  |  |  |  | 2 |
| *RNF213* |  |  |  |  |  | 2 |
| *CREBBP* |  |  |  |  |  | 2 |
| *JAK1* |  |  |  |  |  | 2 |
| *PTPRS* |  |  |  |  |  | 2 |
| *EP300* |  |  |  |  |  | 2 |
| *FANCD2* |  |  |  |  |  | 2 |
| *PIK3CG* |  |  |  |  |  | 2 |
| *PRKDC* |  |  |  |  |  | 2 |
| *ROBO1* |  |  |  |  |  | 2 |
| *COL1A1* |  |  |  |  |  | 2 |
| *IRS2* |  |  |  |  |  | 2 |
| *LATS1* |  |  |  |  |  | 2 |
| *IRS1* |  |  |  |  |  | 2 |
| *PAX7* |  |  |  |  |  | 2 |
| *BCL11A* |  |  |  |  |  | 2 |
| *TET2* |  |  |  |  |  | 2 |
| *CYLD* |  |  |  |  |  | 2 |
| *DNMT3B* |  |  |  |  |  | 2 |
| *STAT4* |  |  |  |  |  | 2 |
| *NKX2-1* |  |  |  |  |  | 2 |
| *NCOA1* |  |  |  |  |  | 2 |
| *DTX1* |  |  |  |  |  | 2 |
| *FANCA* |  |  |  |  |  | 2 |
| *FOXA1* |  |  |  |  |  | 2 |
| *NFATC2* |  |  |  |  |  | 2 |
| *PIK3CD* |  |  |  |  |  | 2 |
| *PMS2* |  |  |  |  |  | 2 |
| *PTPN6* |  |  |  |  |  | 2 |
| *CRTC1* |  |  |  |  |  | 2 |
| *RUNX2* |  |  |  |  |  | 2 |
| *CBL* |  |  |  |  |  | 2 |
| *INPP5D* |  |  |  |  |  | 2 |
| *INSR* |  |  |  |  |  | 2 |
| *JARID2* |  |  |  |  |  | 2 |
| *NFKB2* |  |  |  |  |  | 2 |
| *MERTK* |  |  |  |  |  | 2 |
| *SETD5* |  |  |  |  |  | 2 |
| *ASXL1* |  |  |  |  |  | 2 |
| *CSF1* |  |  |  |  |  | 2 |
| *CSF3R* |  |  |  |  |  | 2 |
| *HOXA3* |  |  |  |  |  | 2 |
| *IFNGR1* |  |  |  |  |  | 2 |
| *PAX3* |  |  |  |  |  | 2 |
| *PBX1* |  |  |  |  |  | 2 |
| *PDCD1* |  |  |  |  |  | 2 |
| *EZR* |  |  |  |  |  | 2 |
| *FGF23* |  |  |  |  |  | 2 |
| *SPOP* |  |  |  |  |  | 2 |
| *SOCS2* |  |  |  |  |  | 2 |
| *CARM1* |  |  |  |  |  | 2 |
| *ARAF* |  |  |  |  |  | 2 |
| *ETV4* |  |  |  |  |  | 2 |
| *EWSR1* |  |  |  |  |  | 2 |
| *FES* |  |  |  |  |  | 2 |
| *HOXD13* |  |  |  |  |  | 2 |
| *HSP90AA1* |  |  |  |  |  | 2 |
| *IRF1* |  |  |  |  |  | 2 |
| *TRAF2* |  |  |  |  |  | 2 |
| *TSHR* |  |  |  |  |  | 2 |
| *RECQL4* |  |  |  |  |  | 2 |
| *HOXB13* |  |  |  |  |  | 2 |
| *ARID5B* |  |  |  |  |  | 2 |
| *MSI2* |  |  |  |  |  | 2 |
| *DDR1* |  |  |  |  |  | 2 |
| *CD36* |  |  |  |  |  | 2 |
| *INHA* |  |  |  |  |  | 2 |
| *LMNA* |  |  |  |  |  | 2 |
| *PDK1* |  |  |  |  |  | 2 |
| *PTPN2* |  |  |  |  |  | 2 |
| *RAD51C* |  |  |  |  |  | 2 |
| *TRAF3* |  |  |  |  |  | 2 |
| *TNFRSF11A* |  |  |  |  |  | 2 |
| *FSTL3* |  |  |  |  |  | 2 |
| *BRD4* |  |  |  |  |  | 2 |
| *DAZAP1* |  |  |  |  |  | 2 |
